# Metabolic Characteristics and M2 Macrophage Infiltrates in Invasive Nonfunctioning Pituitary Adenomas

**DOI:** 10.3389/fendo.2022.901884

**Published:** 2022-07-08

**Authors:** Kunzhe Lin, Jianping Zhang, Yinghong Lin, Zhijie Pei, Shousen Wang

**Affiliations:** ^1^ Department of Neurosurgery, Affiliated Fuzhou First Hospital of Fujian Medical University, Fuzhou, China; ^2^ Fuzong Clinical Medical College of Fujian Medical University, Fuzhou, China; ^3^ Department of Urology, 910th Hospital of Joint Logistics Support Force, Quanzhou, China; ^4^ College of Integrated Chinese and Western Medicine, Fujian University of Traditional Chinese Medicine, Fuzhou, China; ^5^ Department of Neurosurgery, 900th Hospital, Fuzhou, China

**Keywords:** nonfunctioning pituitary adenomas, metabolomics, macrophages, invasive, immunohistochemical

## Abstract

**Objective:**

The aim of this study was to investigate the metabolic differences between invasive and non-invasive nonfunctioning pituitary adenomas (NFPAs), determine the expression of an M2 macrophage marker in NFPAs, and analyze the effects of metabolic changes in invasive NFPAs on M2 macrophage infiltrates.

**Methods:**

Tissue samples of NFPAs from patients who underwent transsphenoidal or craniotomy surgery from January 2021 to August 2021 were collected. NFPA tissues were analyzed based on a gas chromatography-mass spectrometry non-targeted metabolomics platform, and immunohistochemical staining for M2 macrophage marker CD206 was performed.

**Results:**

We evaluated 15 invasive and 21 non-invasive NFPAs. A total of 22 metabolites were identified through non-targeted metabolomics analysis. Among them, the expression of 1-octadecanol, inosine 5’-monophosphate, adenosine 5’-monophosphate, guanosine 5’-monophosphate, creatinine, desmosterol, taurine, hypotaurine, lactic acid, and succinic acid was upregulated in invasive NFPAs, while that of 1-oleoylglycerol, arachidonic acid, cis-11-eicosenoic acid, docosahexaenoic acid, glyceric acid, hypoxanthine, linoleic acid, lysine, oleic acid, uracil, valine, and xanthine was downregulated. Immunohistochemical analysis suggested that the number of CD206-positive cells was higher in invasive NFPAs than in non-invasive NFPAs.

**Conclusion:**

Invasive and non-invasive NFPAs showed distinct metabolite profiles. The levels of succinic acid and lactic acid were higher in invasive NFPAs, and the high expression of the M2 macrophage marker was verified in invasive NFPAs.

## Introduction

Pituitary adenoma is the second most common intracranial tumor, with an incidence of 15% (1). Although they are benign, nearly 35–40% of pituitary adenomas are invasive (2, 3). These adenomas are large; invade the cavernous sinus, sphenoid sinus, and clivus; are prone to recurrence after surgery; and are resistant to conventional treatments, resulting in a more difficult treatment regimen (2, 4). Moreover, the six-year post-surgical survival rate is significantly lower in patients with invasive adenomas than in those with non-invasive pituitary adenomas (5). Therefore, understanding the mechanisms involved in pituitary adenoma invasion could lead to the discovery of new therapeutic targets in the future.

Metabolomics is one of the newest “omics” sciences, evaluating small molecules with molecular masses <1500 Da in various biological fluids or tissues to find a potential correlation of levels of these molecules with the physiological or pathological state of an organism (6). For example, metabolic alterations are characteristic of tumors and promote tumor progression (7, 8). In solid tumors, the microenvironment is often immunosuppressive presenting with hypoxic zones, which induce the metabolic reprogramming in tumor cells from oxidative phosphorylation to anaerobic glycolysis. This glycolytic switch enables hypoxic tumor cells to survive and proliferate (9). Moreover, lysophosphatidylethanolamine metabolism varies among different grades of gliomas: the level of lysophosphatidylethanolamines is decreased, whereas that of short-chain acylcarnitines is increased in high-grade gliomas (10). The related important metabolic pathways associated with glioma progression may provide clues for further studies on the mechanism and treatment of gliomas. Similarly, metabolomic studies on pituitary diseases are still in the early stages (6). Feng et al. have reported metabolic alterations in different pituitary adenoma subtypes (11); however, the metabolic differences between invasive and non-invasive pituitary adenomas remain unclear.

Studies have shown that tumor metabolism produces aberrant metabolites or intermediates that may play important roles in regulating immune cell proliferation, differentiation, activation, and function (12). For example, the metabolites succinate and lactate can promote the polarization of macrophages to the M2 type and promote tumor cell invasion (5, 9, 13). Macrophages respond to various microenvironmental signals generated by tumor and stromal cells, resulting into the alteration of their functional phenotype. Thus, macrophages can be divided into two categories according to their function: classic M1 macrophages and alternative M2 macrophages. M1 macrophages are involved in inflammatory responses, pathogen clearance, and antitumor immunity. Conversely, M2 macrophages influence anti-inflammatory responses, wound healing, and pro-tumor properties (14). Lu et al. have determined the expression of CD68 (a pan-macrophage marker) in macrophages in pituitary adenoma tissue (15); however, the expression of CD68 was not studied in M2 macrophages. Moreover, Yeung et al. investigated the expression of M2 macrophage markers in most pituitary adenomas (16). However, whether the expression of M2 macrophage markers differs between invasive and non-invasive nonfunctioning pituitary adenomas (NFPAs) has not been reported.

The aim of this study was to analyze NFPA tissues based on a gas chromatography (GC)-mass spectrometry (MS) non-targeted metabolomics platform and evaluate the metabolic differences between invasive and non-invasive NFPAs. In addition, CD206 immunohistochemical staining was performed on NFPAs to provide a new target for the treatment of invasive NFPAs.

## Methods

### Patients and Samples

Thirty-six NFPA tissue samples from patients who underwent transsphenoidal or craniotomy surgery at Fuzong Clinical Medical College of Fujian Medical University from January 2021 to August 2021 were collected. During the operation, we used tumor forceps to randomly collect solid tumors. A portion of the fresh tumor sample was frozen at -80°C for metabolomic assays, and a portion was fixed in 10% buffered formalin and embedded in paraffin for immunohistochemical staining. The inclusion criteria were as follows: patients 1) who underwent NFPA resection for the first time and 2) with pathologically confirmed pituitary adenoma. Exclusion criteria were as follows: 1) pituitary adenoma combined with other lesions in the sellar region, such as Rathke cleft cysts and 2) insufficient sample collection. Lesions of Knosp grades 0–2 were defined as non-invasive pituitary adenomas, while those of Knosp grades 3 or 4 were defined as invasive pituitary adenomas (17). Imaging assessment of NFPAs was performed by a neurosurgeon and a neuroradiologist. The mean age of the 36 patients was 49.8 ± 12.6 years. Additionally, 15 NFPAs were invasive, and 21 were non-invasive. The mean volume of the non-invasive NFPAs was 6.1 ± 4.4 cm^3^ and that of the invasive NFPAs was 21.7 ± 17.5 cm^3^(**Table 1**). The study was approved by the Ethics Committees of Fuzong Clinical Medical College of Fujian Medical University. All participants provided written informed consent.

**Table 1:** Patient’s characteristics in invasive and non-invasive NFPA.

Factors	Invasive NFPA (n=15)	Non-invasive NFPA (n=21)
Age, yrs	53.1 ± 13.4	47.5 ± 12.3
Sex		
Male	9	15
Female	6	6
Tumor volume (cm^3^)	21.7 ± 17.5	6.1 ± 4.4
Knosp grades		
0	0	4
1	0	8
2	0	9
3	8	0
4	7	0

NFPA, nonfunctioning pituitary adenomas.

### Metabolomics Analysis

Pituitary tumor tissue (50 mg) was added to an Eppendorf tube containing 1000 μL of chloroform/methanol/water solvent (v/v/v=2:5:2). The pituitary tumor tissue was homogenized in an ice bath using a TissueLyser (JX-24, Jingxin, Shanghai, China) with zirconia beads for 3 min at 30 Hz. A total of 800 μL of supernatant was transferred. The extraction was repeated by adding another 500 μL of ice-cold methanol to the residue. Then, 80 μL of the combined supernatants from the two extractions was mixed with 10 μL of internal standards (0.05 mg/mL of 13C3-15N-L-alanine, 13C5-15N-L-valine, 13C6-L-leucine, and 13C6-15N L-isoleucine) and evaporated to dryness under a gentle nitrogen stream. The dried residue was reconstituted in 30 μL of 20 mg/mL methoxyamine hydrochloride pyridine and incubated at 37°C for 90 min. Following the supplementation of another 30 μL of 1% trimethylchlorosilane, the sample was derivatized at 70°C for 60 min prior to GC-MS metabolomics analysis. Instrumental analysis was performed on an Agilent 7890A/5975C GC-MS system (Agilent Technologies Inc., Santa Clara, CA, USA). An HP 5 MS Accent fused silica capillary column (30 m × 0.25 mm × 0.25 μm; Agilent J&W Scientific, Folsom, CA, USA) was utilized to separate the derivatives.

For multivariate statistical analysis, the normalized data were preprocessed by UV scaling and mean centering before performing principal component analysis (PCA), partial least squares-discriminant analysis (PLS-DA), and orthogonal projections to latent structures-discriminant analysis (OPLS-DA). For univariate statistical analysis, the normalized data were calculated using Student’s t-test.

The factors with variable importance in the projection (VIP) values in the OPLS-DA model >1 and *p*-values from univariate statistical analysis <0.05 were identified as potential differential metabolites. Fold change (log2FC) was calculated as a binary logarithm of the average normalized peak intensity ratio between invasive and non-invasive NFPAs, where positive values indicated that the average mass response of invasive NFPAs was higher than that of non-invasive NFPAs. Metabolomics pathway analysis of the metabolic biomarkers was carried out using MetaboAnalyst (Version 5.0, https://www.metaboanalyst.ca).

### Immunohistochemical Analysis

Immunohistochemical staining was performed on paraffin-embedded NFPA tissues. The tissues were sectioned with a thickness of 4 µm, and the sections were deparaffinized in xylene washing solution three times, followed by rehydration with ethanol. The sections were then immersed in citrate retrieval solution and heated in a microwave oven for antigen retrieval. Then, the sections were incubated in 3% H_2_O_2_ for 10 min to inhibit endogenous peroxidase activity and washed three times in phosphate-buffered saline (PBS). The sections were incubated with primary antibody rabbit anti-human CD206 monoclonal antibody (1:400, 91992S, CST) overnight at 4°C. The sections were washed thrice with PBS to remove antibody solution. They were then incubated with secondary antibody (1:50,000; KIT-5010; anti-rabbit/mouse IgG; Maixin Biotechnology Development Co., Ltd., Fuzhou, China) for 30 min at 37°C and washed with PBS. Next, the sections were stained with 3,3’-diaminobenzidine and a substrate-chromogen for 2 min at room temperature and redyed with hematoxylin. The stained tissue sections were observed using a CX41 microscope (Olympus, Tokyo, Japan) and assessed by two pathologists. Positively stained cells were counted in every field under high magnification (400×). The mean number from three random fields was used for analysis of each patient.

## Statistical Analysis

The number of CD206-positive cells in the NFPA samples was expressed as the mean ± standard deviation. Comparisons between two groups were performed using a two-tailed unpaired Student’s t-test (for parametric analysis) or Mann–Whitney U test (for non-parametric analysis). Statistical analysis was performed using SPSS 19 (SPSS Inc., Chicago, IL, USA) and GraphPad Prism 5.0 (GraphPad Software, Inc., San Diego, CA, USA). *P*-values <0.05 were considered statistically significant.

## Results

### Differential Metabolite Screening

**Figure 1:**
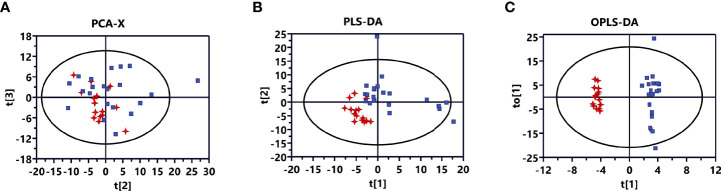
Multivariate statistical analysis for invasive nonfunctioning pituitary adenoma (NFPA) and non-invasive NFPA tissues. **(A)** Principal component analysis (PCA) score plot based on all differential metabolites for invasive (red dots) and non-invasive (blue dots) NFPA tissues. **(B)** Partial least squares-discriminant analysis (PLS-DA) score plot based on all differential metabolites for invasive (red dots) and non-invasive (blue dots) NFPA tissues. **(C)** Orthogonal projections to latent structures-discriminant analysis (OPLS-DA) score plot based on all differential metabolites for invasive (red dots) and non-invasive (blue dots) NFPA tissues.

A plot of the PCA scores from the entire metabolite dataset did not show separations (**Figure 1A**). However, the plot of the PLS-DA scores demonstrated clear separations between invasive and non-invasive NFPAs (**Figure 1B**). Moreover, the OPLS-DA model could effectively distinguish the two groups of samples from invasive and non-invasive NFPAs (**Figure 1C**). The main quality parameters of the model were R^2^X=0.629, R^2^Y=0.984, and Q^2 =^ 0.528.

Twenty-two metabolites with VIP >1 and *P <*0.05 were differentially expressed between invasive and non-invasive NFPAs. Among them, the expression of 12 metabolites downregulated, while that of 10 metabolites upregulated. The metabolites with upregulated expression in invasive NFPA were 1-octadecanol, inosine 5′-monophosphate (IMP), adenosine 5’-monophosphate (AMP), guanosine 5’-monophosphate (GMP), creatinine, desmosterol, taurine, hypotaurine, lactic acid, and succinic acid, while the metabolites with downregulated expression were 1-oleoylglycerol, arachidonic acid, cis-11-eicosenoic acid, docosahexaenoic acid (DHA), glyceric acid, hypoxanthine, linoleic acid, lysine, oleic acid, uracil, valine, and xanthine. The data of the differential metabolites are shown in **Table 2**.

**Table 2:** The metabolic differences between invasive and non-invasive NFPA.

Metabolite	VIP	P-value	Log2FC (A/B)	HMDB	KEGG
1-Octadecanol	1.80	2.59E-03	0.57	HMDB0002350	D01924
1-Oleoylglycerol	1.47	2.06E-02	-0.51	HMDB0011567	–
AMP	1.73	3.02E-03	0.97	HMDB0000045	C00020
Arachidonic acid	1.53	1.53E-02	-1.28	HMDB0001043	C00219
cis-11-Eicosenoic acid	1.63	6.34E-03	-1.23	HMDB0002231	C16526
Creatinine	1.34	2.74E-02	1.17	HMDB0000562	C00791
Desmosterol	1.38	1.91E-02	0.86	HMDB0002719	C01802
DHA	1.47	2.49E-02	-1.10	HMDB0002183	C06429
Glyceric acid	1.29	4.64E-02	-1.58	HMDB0000139	C00258
GMP	2.07	8.13E-05	1.29	HMDB0001397	C00144
Hypotaurine	1.34	2.57E-02	1.35	HMDB0000965	C00519
Hypoxanthine	1.48	2.32E-02	-0.56	HMDB0000157	C00262
IMP	1.60	4.44E-03	1.53	HMDB0000175	C00130
Lactic acid	1.47	1.70E-02	0.23	HMDB0000190	C00186
Linoleic acid	1.89	1.13E-03	-0.83	HMDB0000673	C01595
Lysine	1.43	3.76E-02	-0.53	HMDB0000182	C00047
Oleic acid	1.80	2.60E-03	-1.17	HMDB0000207	C00712
Succinic acid	3.09	1.94E-14	2.05	HMDB0000254	C00042
Taurine	1.39	1.96E-02	0.38	HMDB0000251	C00245
Uracil	1.82	2.07E-03	-1.34	HMDB0000300	C00106
Valine	1.42	3.03E-02	-0.48	HMDB0000883	C00183
Xanthine	1.22	4.34E-02	-1.32	HMDB0000292	C00385

NFPA, nonfunctioning pituitary adenomas; AMP, adenosine 5′-monophosphate; IMP, inosine 5′-monophosphate; GMP, guanosine 5’-monphosphate; DHA, Docosahexaenoic acid; VIP, Variable Importance in the Projection, was obtained from the OPLS-DA model. The p value was calculated from Student’s t test. Log2FC, fold change, was calculated as a binary logarithm of the average mass response (normalized peak area) ratio between Group **A** (invasive NFPA) vs Group **B** (non-invasive NFPA), where a positive value means that the average mass response of the metabolite in Group **A** is larger than that in Group **B.**

### Pearson Correlation and Heatmap Analyses

**Figure 2:**
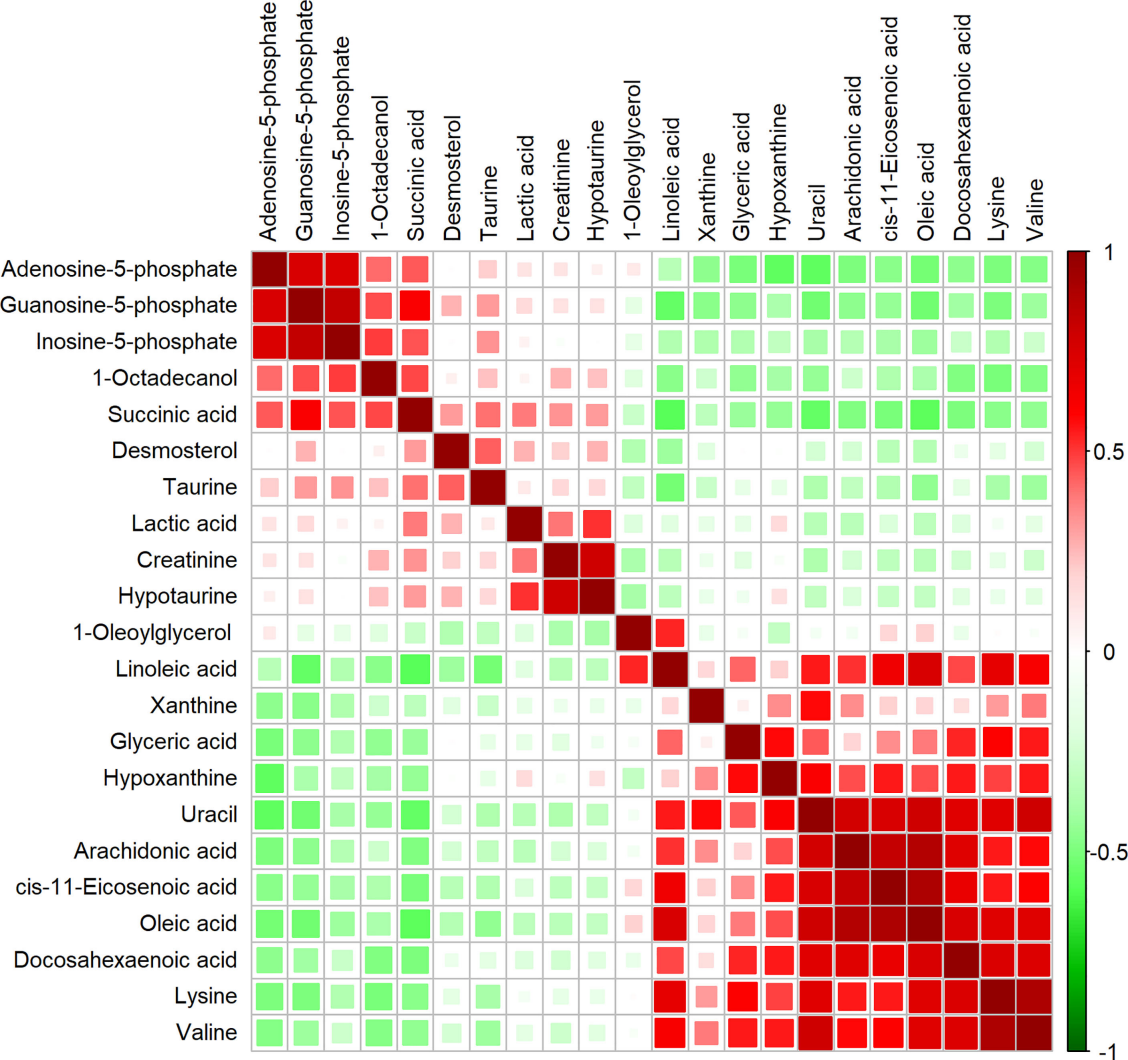
Correlation matrix obtained from significantly altered metabolites between invasive nonfunctioning pituitary adenoma (NFPA) and non-invasive NFPA tissues. Differential metabolites are represented in each row and column. The correlation coefficient values are shown on the right side of the figure. The size and color of the squares in the figure are related to the correlation between the differential metabolites. Red indicates a positive correlation between differential metabolites, and green indicates a negative correlation between differential metabolites.

To characterize the quantitative correlation between the different metabolites, we performed Pearson correlation analysis (R platform, version 3.4.3) on the quantitative data of these metabolites. Pearson correlation analysis demonstrated a positive correlation among IMP, AMP, and GMP. Arachidonic acid, DHA, oleic acid, and cis-11-eicosenoic acid were also positively correlated (**Figure 2**).

**Figure 3:**
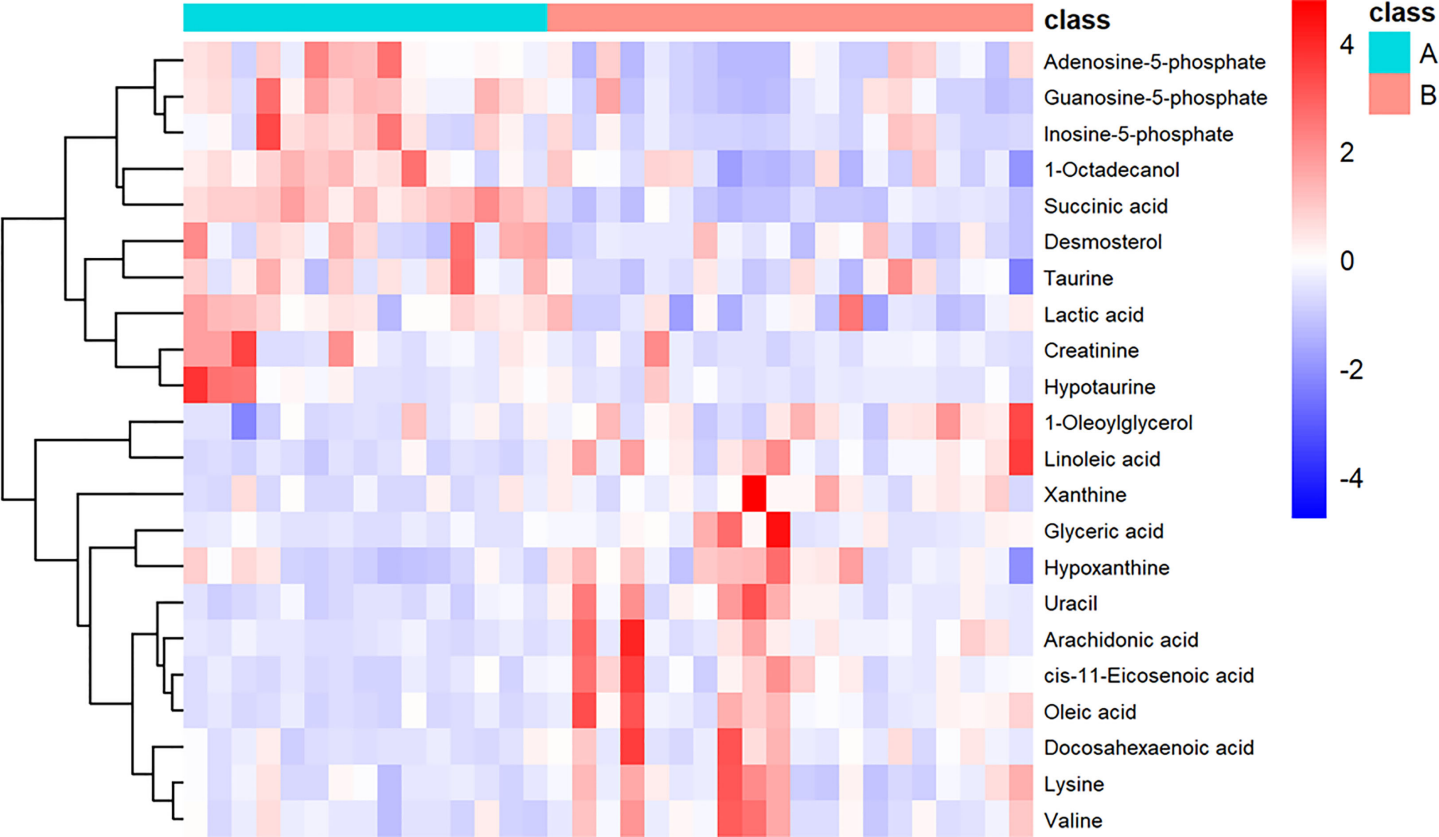
Heatmap showing the 22 important metabolites for invasive nonfunctioning pituitary adenoma (NFPA) and non-invasive NFPA tissues. Class A (invasive NFPA), Class B (non-invasive NFPA).

We also performed heatmap analysis (R platform, version 3.4.3) on the quantitative information of these differential metabolites (**Figure 3**). The results showed metabolites with upregulated and downregulated expression in invasive NFPA, similar to those previously described.

### Pathway Analysis

**Figure 4:**
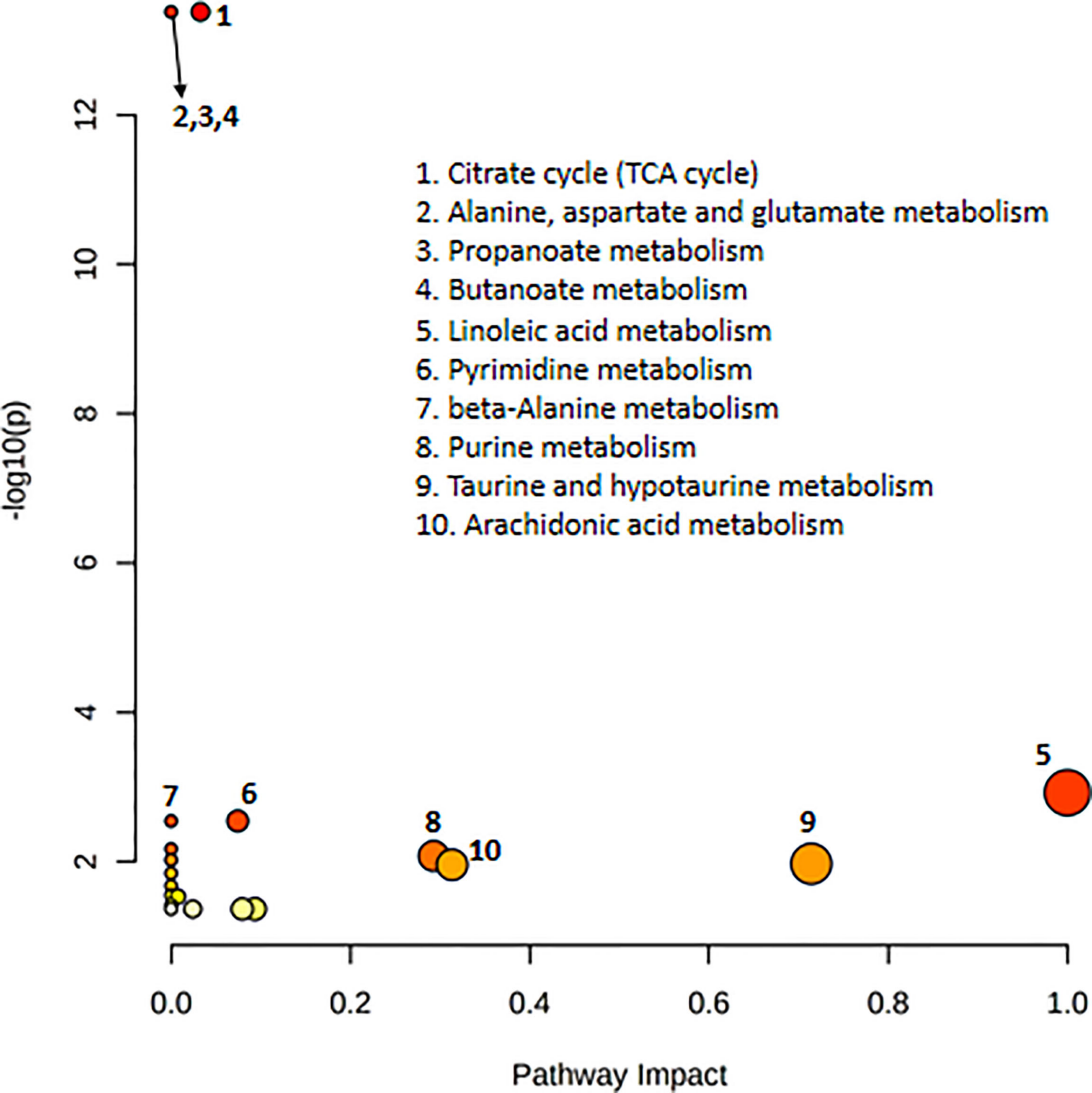
Pathway topology analysis depicting the dysregulated metabolic pathways associated with invasive nonfunctioning pituitary adenoma NFPA. The node color represents the *p*-values from pathway enrichment analysis, and the node radius indicates the pathway impact values.

Pathway analysis of differential metabolites was performed using MetaboAnalyst; the metabolome view is shown in **Figure 4**. Metabolic pathways associated with NFPA aggressiveness included the tricarboxylic acid cycle, as well as alanine, aspartate, glutamate, propanoate, and butanoate metabolism. The most important metabolic pathways were linoleic acid metabolism as well as taurine and hypotaurine metabolism.

### Infiltration of CD206 Macrophages in NFPA

**Figure 5:**
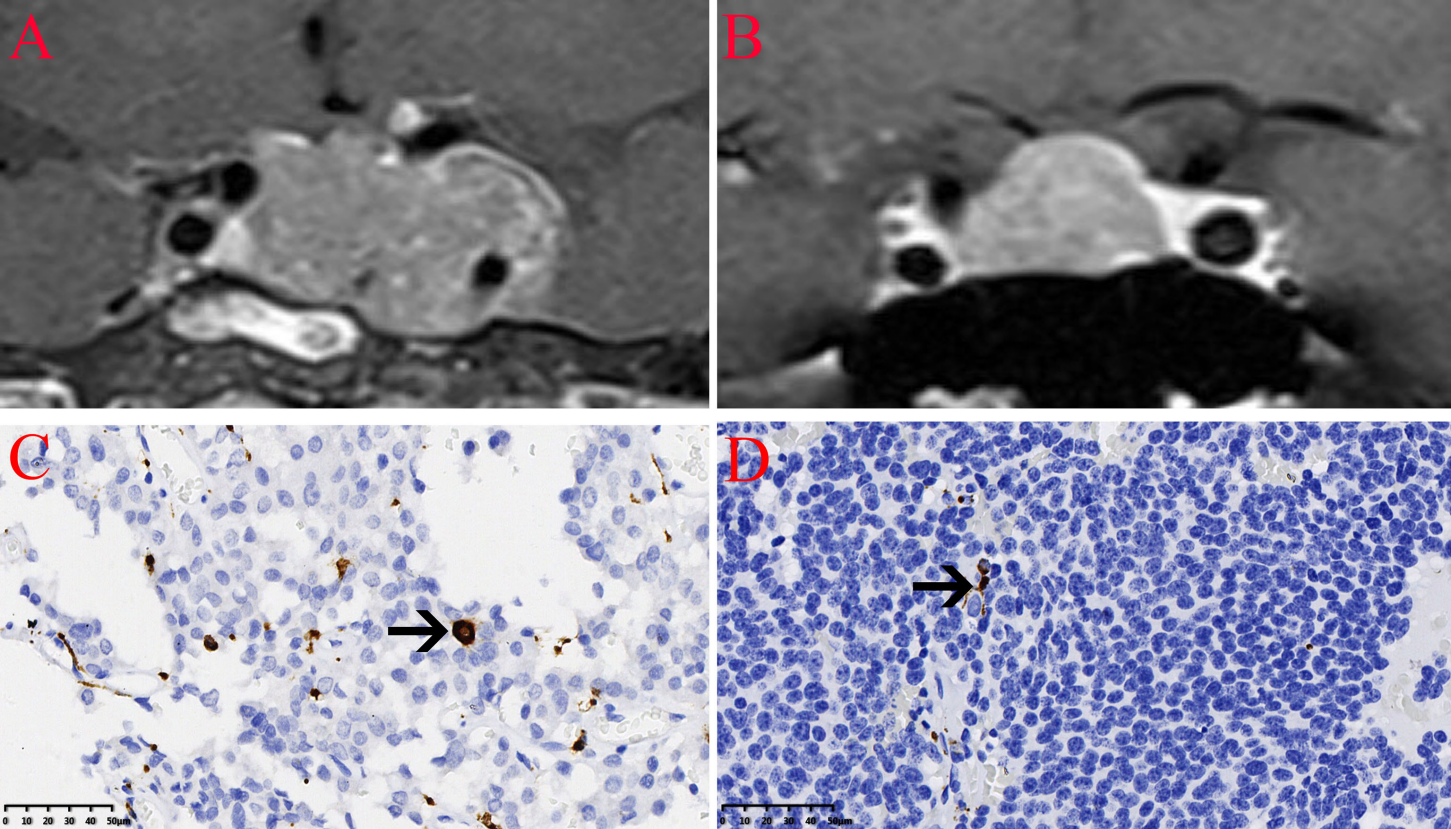
Infiltration of CD206 macrophages in nonfunctioning pituitary adenomas NFPAs. The CD206 macrophages are indicated by the arrow. **(A)** Coronal contrast-enhanced image showing invasive pituitary adenomas. **(B)** Coronal contrast-enhanced image showing non-invasive pituitary adenomas. **(C)** Infiltration of CD206 macrophages in invasive NFPAs.** (D)** Infiltration of CD206 macrophages in non-invasive NFPAs.

**Figure 6:**
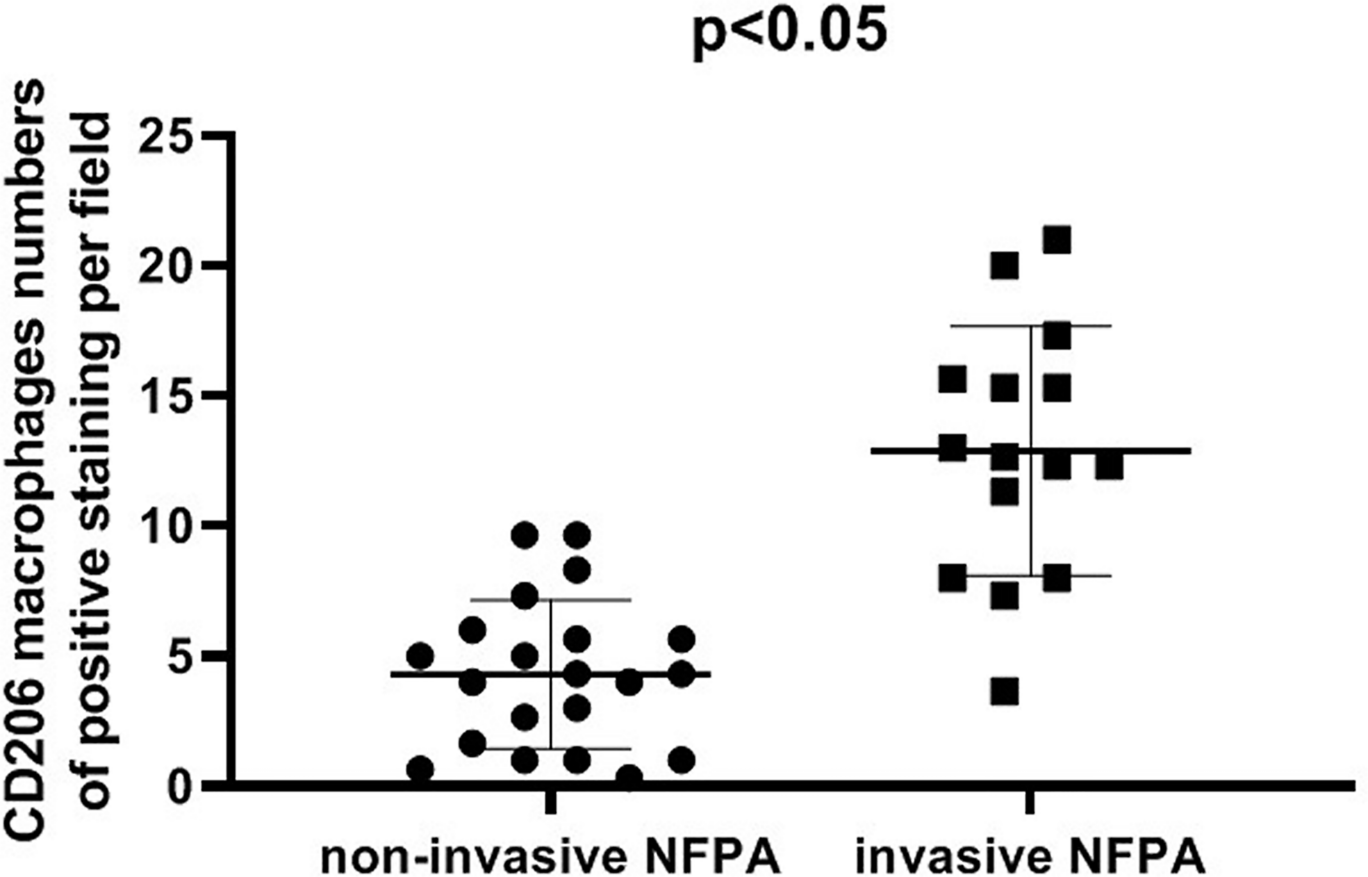
Comparison of CD206 macrophage expression between invasive nonfunctioning pituitary adenoma (NFPA) and non-invasive NFPA.

The expression of the M2 macrophage marker CD206 and presence of M2 macrophages in NFPA were evaluated using immunohistochemistry (**Figure 5**). The number of CD206-positive cells in invasive NFPA (12.66 ± 4.80 cells/field) was higher than that in non-invasive NFPA (4.00 ± 3.02 cells/field, p < 0.001) (**Figure 6**).

## Discussion

Metabolic alterations in pituitary adenomas are poorly understood. In this study, we aimed to understand the metabolic differences between invasive and non-invasive NFPAs and provide a new perspective on the aggressiveness of pituitary adenomas.

This study is the first to identify the accumulation of succinate and lactate in invasive NFPA. Succinic acid is an intermediate metabolite in the tricarboxylic acid cycle, which is a metabolic pathway for the production of cellular energy and biosynthetic intermediates in aerobic organisms (18). The accumulation of succinic acid suggests blockage of the tricarboxylic acid cycle pathway. Moreover, lactic acid is the metabolic end product of glycolysis. In contrast to normal cells, tumor cells tend to undergo glycolysis in the cytoplasm even in the presence of oxygen, which is known as the “Warburg effect” (12). Thus, aerobic oxidative metabolism and glycolytic metabolism differ between invasive and non-invasive NFPAs. Hence, the role of succinic acid and lactic acid in promoting the invasion of pituitary adenomas is yet to be determined.

Studies have shown that high infiltration of macrophages in solid tumors is associated with poor clinical outcomes (19). Multiple components of the tumor microenvironment induce macrophage polarization to the M2 type, thus exerting a tumor-promoting effect. Moreover, cancer-derived succinate has been demonstrated to promote macrophage polarization and cancer metastasis by activating the PI3K-hypoxia-inducible factor 1a axis (13). In addition, Zhang et al. showed that lactate secreted by pituitary adenoma cells promotes macrophage polarization to the M2 type through the mTORC2 and ERK signaling pathways (5). However, the differential expression of M2 macrophage markers in invasive NFPA versus non-invasive NFPA is unknown. In addition to confirming the accumulation of succinate and lactate in invasive NFPA, we also found that the expression of M2 macrophage marker CD206 was significantly upregulated in invasive NFPA. Finally, M2 macrophages mainly promote tumor invasiveness by suppressing immunity, pituitary adenoma cell epithelial-mesenchymal transition and proliferation, vascularization, and extracellular matrix remodeling (20).

The metabolomics analysis in this study suggested that the unsaturated fatty acid biosynthesis and metabolic pathways differed between invasive and non-invasive NFPA, including that of arachidonic acid, DHA, oleic acid, and cis-11-eicosenoic acid. We observed that the levels of the above metabolites were lower in invasive NFPA than in non-invasive NFPA. Arachidonic acid and linoleic acid are omega-6 polyunsaturated fatty acids, which have tumor-promoting effects, while DHA is an omega-3 polyunsaturated fatty acid, which has a tumor-suppressing effect (21). Furthermore, DHA has anti-inflammatory, anti-proliferative, pro-apoptotic, anti-angiogenic, anti-invasive, and anti-metastatic properties (22). Wang et al. compared the differences in the levels of serum polyunsaturated fatty acids between patients with malignant tumors and healthy people. They found that the levels of arachidonic acid, total omega-6, DHA, and total omega-3 were significantly reduced in those with malignant tumors (23). Peroxidation of omega-3 and omega-6 polyunsaturated fatty acids exerts antitumor effects in acidic tumor environments (24). Our study initially revealed a difference in lipid metabolism between invasive and non-invasive NFPAs; however, the association of this difference with the aggressiveness of NFPA warrants further investigation.

Purines are an essential component of nucleotides in cell proliferation (25). Increased purine synthesis may be associated with tumorigenesis, and metabolites of the purine synthesis pathway, such as IMP, AMP, adenine, and GMP, have recently received extensive attention (26). Moreover, the levels of IMP, AMP, and GMP were increased in tumor stem cells (27). The present study revealed that the levels of metabolites in the purine synthesis pathway were higher in invasive NFPA than in non-invasive NFPA, suggesting that purine metabolism is involved in NFPA invasion. In addition, the levels of xanthine and hypoxanthine were decreased in invasive NFPA, which may be due to increased DNA synthesis in hyperproliferative tissues (28). These metabolites are intermediates of the purine degradation pathway.

We found taurine and hypotaurine to be important differential metabolites between invasive and non-invasive NFPAs. Elevated levels of taurine can be seen in breast cancer (29), thyroid cancer (30), colon cancer (31), and acute myeloid leukemia (32). In addition, taurine concentrations in gliomas and meningiomas were higher than those in extra-tumoral brain tissue (33). Increased taurine synthesis is a cellular response to hypoxia and reperfusion injury, as repeated hypoxia leads to higher levels of taurine (34). However, taurine is also thought to exert antitumor effects by inducing tumor cell apoptosis through various mechanisms (35). Nevertheless, the mechanism of action of taurine in invasive NFPAs requires further study. Meanwhile, studies have shown that hypotaurine, an intermediate in the biosynthesis of taurine is positively correlated with glioma grade and promotes cell proliferation and invasion by competitively inhibiting prolyl hydroxylase domain-2, leading to activation of the hypoxia signal (36).

Invasive and non-invasive NFPAs showed distinct metabolite profiles. Alterations in unsaturated fatty acid biosynthesis, as well as tricarboxylic acid, glycolytic, purine, taurine, and hypotaurine metabolism may be involved in the aggressiveness of NFPA.

Our study has several limitations. First, the sample size of this study was relatively small, and further studies with larger sample sizes are needed to confirm the metabolic differences between invasive and non-invasive NFPAs. Second, key differential metabolites determined by metabolomic analysis, such as succinic acid and lactic acid, require further verification by western blotting and quantitative reverse-transcription polymerase chain reaction. Finally, the changes in metabolites affecting macrophage phenotype in pituitary adenomas lack verification at the cellular level.

## Conclusions

In this study, metabolomic analysis was performed on invasive and non-invasive NFPAs, and the differential metabolites and metabolic pathways were preliminarily identified. We verified the high expression of the M2 macrophage marker CD206 in invasive NFPAs by immunohistochemistry.

## Data Availability Statement

The original contributions presented in the study are included in the article/supplementary material. Further inquiries can be directed to the corresponding author.

## Ethics Statement

The studies involving human participants were reviewed and approved by Fuzong Clinical Medical College of Fujian Medical University. The patients/participants provided their written informed consent to participate in this study.

## Author Contributions

KL, JZ, and SW contributed to conception and design of the study. KL and YL performed data collection. JZ and YL performed the statistical analysis. KL wrote the first draft of the manuscript. JZ, YL, ZP and SW wrote sections of the manuscript. All authors contributed to manuscript revision, read, and approved the submitted version.

## Funding

This work was supported by the Natural Science Foundation of Fujian Province [2021J011306] and Department of Science and Technology of Fuzhou City [2021-S-180].

## Conflict of Interest

The authors declare that the research was conducted in the absence of any commercial or financial relationships that could be construed as a potential conflict of interest.

## Publisher’s Note

All claims expressed in this article are solely those of the authors and do not necessarily represent those of their affiliated organizations, or those of the publisher, the editors and the reviewers. Any product that may be evaluated in this article, or claim that may be made by its manufacturer, is not guaranteed or endorsed by the publisher.
